# HIV-1 Vpr N-terminal tagging affects alternative splicing of the viral genome

**DOI:** 10.1038/srep34573

**Published:** 2016-10-10

**Authors:** Ann Baeyens, Evelien Naessens, Anouk Van Nuffel, Karin E. Weening, Anne-Marie Reilly, Eva Claeys, Wim Trypsteen, Linos Vandekerckhove, Sven Eyckerman, Kris Gevaert, Bruno Verhasselt

**Affiliations:** 1 Department of Clinical Chemistry, Microbiology, and Immunology, Ghent University, Ghent, Belgium; 2 HIV Translational Research Unit, Department of Internal Medicine, Faculty of Medicine and Health Sciences, Ghent University and Ghent University Hospital, Ghent, Belgium; 3 VIB Medical Biotechnology Center, B-9000 Ghent, Belgium; 4 Department of Biochemistry, Ghent University, B-9000 Ghent, Belgium

## Abstract

To facilitate studies on Vpr function in replicating HIV-1, we aimed to tag the protein in an infectious virus. First we showed that N-, but not C-terminal HA/FLAG tagging of Vpr protein preserves Vpr cytopathicity. Cloning the tags into proviral DNA however ablated viral production and replication. By construction of additional viral variants we could show this defect was not protein- but RNA-dependent and sequence specific, and characterized by oversplicing of the genomic RNA. Simulation of genomic RNA folding suggested that introduction of the tag sequence induced an alternative folding structure in a region enriched in splice sites and splicing regulatory sequences. *In silico* predictions identified the HA/His_6_-Vpr tagging in HIV-1 to affect mRNA folding less than HA/FLAG-Vpr tagging. *In vitro* infectivity and mRNA splice pattern improved but did not reach wild-type values. Thus, sequence-specific insertions may interfere with mRNA splicing, possibly due to altered RNA folding. Our results point to the complexity of viral RNA genome sequence interactions. This should be taken into consideration when designing viral manipulation strategies, for both research as for biological interventions.

Vpr is a pleiotropic accessory HIV-1 protein, that enhances infection of resting cells, modulates both HIV and cellular transcription, along with induction of G2 arrest and cell death (reviewed by Kogan^1^). Although Vpr is dispensable for *in vitro* infection and replication, *in vivo* studies have shown that infection with Vpr-deleted or mutated HIV/SIV strains are less deleterious to the host^2^. These mutated strains tend to revert to full Vpr function^3^, which is reflected by high sequence conservation of the protein over viral isolates^4^. Given the small genome, HIV-1 maximizes its genetic coding potential by using three reading frames and alternative splicing^5^. Three types of mRNA are expressed: multiple spliced (coding for Tat, Rev and Nef protein), singly spliced (Env, Vif, Vpu and Vpr) and unspliced transcripts (Gag, Pol and the viral genome). Splicing occurs when the intronic sequence between a 5′ splice donor (5′ ss) and a 3′ splice acceptor (3′ ss) is excised by the spliceosome^6^. The recognition of these splice sites is regulated mainly by the intrinsic strength of the splice site, and can be influenced by the presence of polypyrimidine tracts (PPTs) and branch site sequences. Additionally, the virus encodes cis-acting exonic and intronic silencers/enhancers that affect splicing^7^ and typically mutations within these sequences can impact viral replication^8^,^9^,^10^. However, it is becoming clear that not only the sequence of splicing elements can influence the splicing process but also local RNA structures, modulating binding of spliceosome elements and splice site usage^11^,^12^,^13^.

HIV-1’s small genome also implies dependency on cellular host proteins and machinery for viral replication: HIV proteins need to interact with cellular proteins to exert their function^14^. Here, we designed a strategy to identify and evaluate host proteins interacting with Vpr, to study its regulation and function. Protein tagging facilitates pull-down, to screen for and identify protein-protein interactions using mass spectrometry^15^. A dual-tagged Vpr fusion protein approach was chosen, allowing for tandem affinity purification, which has the benefit of reducing non-specific interactions^16^. While fusion proteins have the advantage that the antibody for pull-down does not need to target bait protein epitopes possibly shielded by interaction partners, tagging of proteins can affect their function^17^. Hence, initially the function of amino- (N) versus carboxy- (C) terminal tagged Vpr was compared. Subsequently, tagged Vpr in a replicating HIV-1 was constructed to provide a tool to study protein function in an infectious cycle at biologically relevant protein levels. Our experiments provide new experimental insights not only on HIV-1 viral protein tagging strategies but also on the biology of HIV-1 mRNA splicing.

## Results

### Vpr N-terminal, but not C-terminal, tagging preserves cytopathic function

To reduce possible steric hindrance on protein function, the use of small tags is recommended^18^. We therefore chose a FLAG/HA tandem tag to tag Vpr as a fusion protein. Single glutamine linkers (Q residue) were added between the tags and Vpr to further improve stability and bioactivity of the fusion protein^19^.

**Figure 1:**
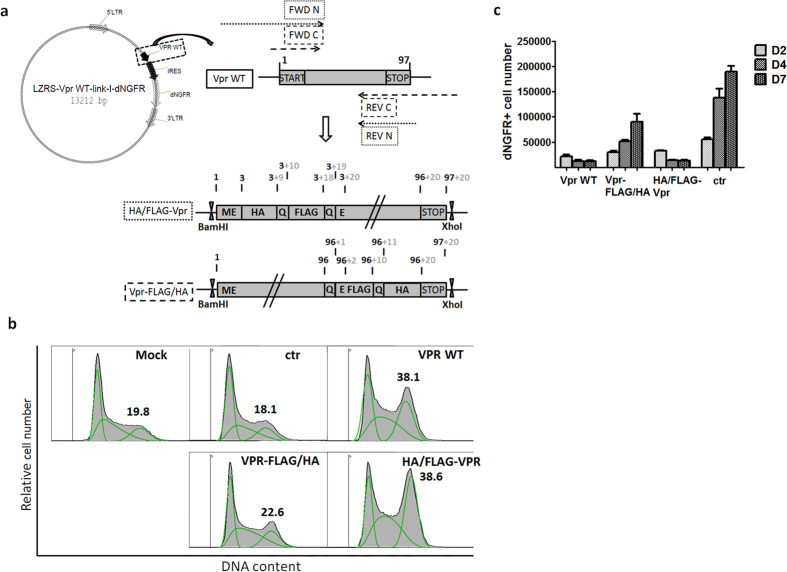
N/C Tagged Vpr cloning and functional testing. (**a**) Molecular cloning strategy for N/C-terminal tagging to Vpr. FWD N and REV C primers have non-binding tails containing the HA and FLAG tags. More detailed methodology can be found in Supplement 1, together with primer sequences. In the final LZRS retroviral vector constructs, the wild-type Vpr or Vpr tagged fusion proteins (either HA/FLAG-Vpr (N-terminal tags) or Vpr-FLAG/HA (C-terminal tags)) are cloned and expressed from a bicistronic mRNA (IRES sequence) also encoding dNGFR as a separate marker protein to identify construct expressing cells. Both tagged proteins are preceded by Met-Glu (ME) and HA, FLAG and Vpr are separated by single Gln (Q) linkers. Numbers indicate amino acid position of Vpr (black), and number of amino acids added by tagging (grey). (**b**) Cell cycle analysis on pLZRS-transfected 293T cells, representative of one of three experiments. 48 h post-transfection, cells were harvested, sorted for dNGFR expression, RNase A treated and stained with propidium iodide for cellular DNA content. Control samples were either untransfected (Mock) or transfected with a control LZRS vector (ctr). Data were analyzed with the FlowJo cell cycle analysis platform, applying the Dean-Jett-Fox model. Separate green curves in the shaded histogram illustrate the fraction of cells in G1, S and G2 phase of the cell cycle. The percentage of cells in G2 phase (represented by green curve covering highest DNA content) is shown for each sample. (**c**) Absolute number of dNGFR marker positive SupT1 cells, in cultures after 2 (D2), 4 (D4 or 7 (D7) days after transduction with LZRS retroviral particles. Three different Vpr constructs were transduced as indicated, or a control LZRS vector was used (ctr).

First, we determined whether N- or C-terminal tagging of Vpr was most appropriate to preserve cytopathicity, defined by a combination of cytotoxic and cytostatic activity. For this, tagged Vpr was cloned into a retroviral vector that allows for expression from a bicistronic mRNA also encoding dNGFR using a PCR strategy as shown in Fig. 1a. The resulting construct was transfected in 293T cells to assess Vpr induced G2 cell cycle arrest and survival. As shown in Fig. 1b, N-terminal tagging (HA/FLAG-VPR) conserved cell cycle arrest induction comparable to that by the wild-type (WT) Vpr while the C-terminal tagged Vpr (VPR-FLAG/HA) lost this property. We also observed that only N-terminal tagging preserves the cytopathic functions of Vpr: in Fig. 1c, the fraction of dNGFR marker expressing SupT1 cells over time is presented, as an indirect assessment of Vpr’s cytopathic effect. While Vpr expressing cells regressed throughout culture, control transduced cells grew continuously. The phenotype of HA/FLAG-Vpr resembles that of the WT protein, unlike C-terminal Vpr-FLAG/HA transduced cells, exhibiting slightly reduced growth. Together, we conclude from these data that only N-terminal tagging of Vpr preserves cytopathic function. In the scope of this study, we therefore selected N-terminal HA/FLAG-Vpr tagging strategy to be applied in HIV-1.

### HIV-1 Vpr HA/FLAG N-terminal tagging abrogates viral production and replication

**Figure 2:**
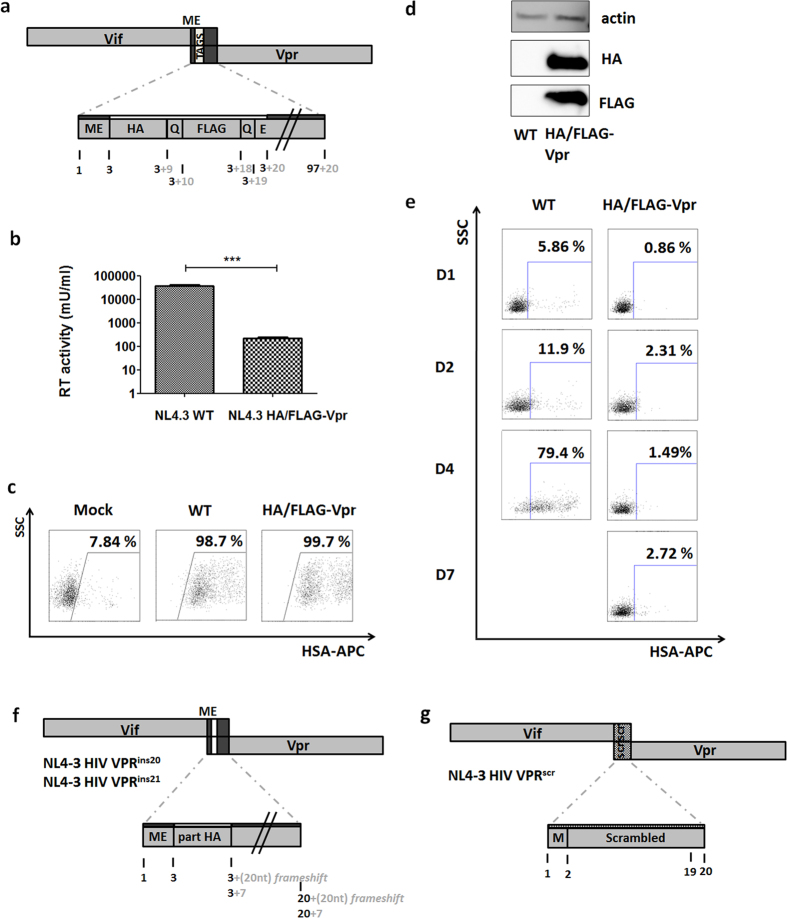
pNL4-3 HIV-1 HA/FLAG-Vpr cloning and functional testing. (**a**) Representation of HIV-1 reading frames 1 (Vif) and 3 (Vpr) showing the overlap. The zoomed detail displays the composition of the Vpr protein N-terminus, containing HA and FLAG tags, preceded by Met-Glu (ME), and separated by single Gln (Q) linkers. Numbers indicate amino acid position of Vpr (black), and number of amino acids added by tagging (grey) (**b**) Reverse Transcriptase (RT) activity of 293T-produced virus. 48 h post-transfection, viral supernatant was harvested and quantified for RT activity. N = 14, Mann-Whitney U test, p < 0.0001. (**c**) HSA expression of pNL4-3 HIV-1 HA/FLAG-Vpr plasmid transfected 293T cells measured by flow cytometry after staining with an APC labelled antibody, 48 h post-transfection as marker of transfection efficiency. Dot plots show HSA-APC staining versus side scatter (SSC). Numbers indicate percentage of cells staining positively. (**d**) Western blot for HA and FLAG expression of pNL4-3 HIV-1 WT or HA/FLAG-Vpr transfected 293T cells, 48 h post-transfection, β-actin was stained as loading control. (**e**) SupT1 infection, quantified by HSA expression measured by flow cytometry on different time points post-infection (D1 to D7 as indicated). Cells were infected with 40 ng p24 WT virus or, due to defective viral production, 100 μl HA/FLAG-Vpr viral supernatant. At day 7 post-infection WT infected cells had succumbed. Numbers indicate percentage of cells staining positively. (**f**) Representation of the VPR^ins20^ and VPR^ins21^ mutant in the HIV-1 reading frames 1 (Vif) and 3 (Vpr). The overlap of both reading frames is shown, as well as 20 or 21 bp inserted from HA. Numbers indicate amino acid position of Vpr (black), and number of amino acids added by the insert (grey), in the case of VPR^ins20^ the frame shift induced does not allow to count amino acids added (**g**) Simplified representation of the VPR^scr^ mutant in the HIV-1 reading frames 1 (Vif) and 3 (Vpr). The overlap of both reading frames is replaced with a scrambled sequence, but without losing the ATG start codon or without introducing premature stop codons. Numbers indicate amino acid position of the artificial protein (black).

To create the HIV-1 HA/FLAG-Vpr construct, an overlap extension PCR was applied to incorporate tags, as described in Supplement 1. The resulting construct is shown in Fig. 2a.

To ensure efficient and stable expression of the HA/FLAG-Vpr fusion protein, a Met-Glu amino acid doublet was positioned upstream of the tag, since we found this conserved doublet to be important for post-translational stability of Vpr (unpublished data). Since Vif and Vpr share a 60 base-pairs overlap, inserting the tags accordingly results in an insertion in Vif what could affect function. To exclude this potential bias, infection experiments were done in cells (293T and SupT1) permissive for HIV lacking functional Vif^20^. Unexpectedly, when using this proviral construct to produce virions, viral production was nearly ablated (Fig. 2b). Expression of the virus encoded marker gene (HSA) from the HSA-IRES-nef reading frame was not reduced (Fig 2c), indicating transfection of proviral plasmid was efficient. The HA/FLAG-Vpr fusion protein could be demonstrated on Western blot (Fig. 2d). The viruses produced were strongly affected in replication in SupT1 cells, as shown in Fig. 2e, using the HSA marker to quantify infection. This reduced replication was confirmed in CEM-GFP reporter cells, encoding a GFP marker protein in response to LTR transcriptional activity, thus a different cell line and a different reading of viral expression (Supplement 2).

### Effect of Vpr N terminus insertions is RNA based and sequence specific

To rule out that these observations were due to a particular property of the HIV-1 HSA-IRES-Nef reporter virus, the tags were cloned into a WT HIV-1 backbone (Supplement 3). We could reproduce the inhibition of viral production, confirming that the inserted tags sequence cause impaired viral production and replication even in a WT virus not modified to express a marker gene.

Next, we assessed if other, smaller insertions at the same position could have the same outcome and whether reading frames are involved. Therefore, we first inserted either a 20 or 21 base-pair long insert in the N-terminus of Vpr (VPR^ins20^ and VPR^ins21^ constructs respectively, Fig. 2f; cloning is described in Supplement 1), similar but shorter compared to the modification by HA/FLAG-Vpr tagging (60 basepairs). Only a N-terminal fragment of the original HA insertion of 21 or 20 bp length was inserted, so respectively conserving or shifting the Vif and Vpr reading frame.

We found that both constructs did not affect viral production nor infection (Supplement 4), thus disruption of the corresponding protein domains did not explain the observation and shorter insertions are insufficient to affect viral fitness. Next we asked whether changes in the local sequence was sufficient to induce viral defects. To test this, a 60 bp scrambled sequence replacing the WT sequence (VPR^scr^, Fig. 2g; cloning is described in Supplement 1) was cloned into the Vif/Vpr overlap. We preserved the start codon of Vpr and ruled out the introduction of stop codons. Similar to VPR^ins20^ or VPR^ins21^, both viral production and infection were unaffected. (Supplement 4).

Together, from these mutants we conclude that the effect of Vpr N terminus insertions is not due to alterations in Vpr or Vif protein, and sequence specific.

### Viral defects following Vpr N terminus insertions correlate with oversplicing

**Figure 3:**
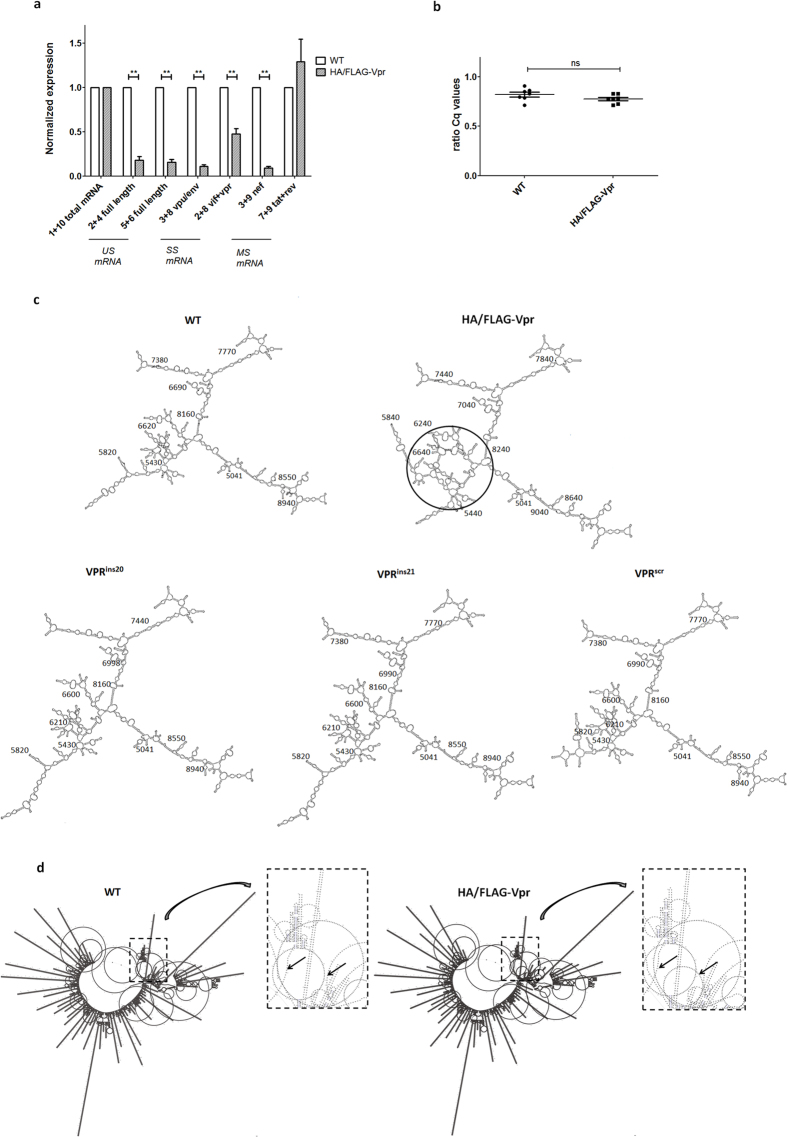
HIV-1 HA/FLAG-Vpr splicing and mRNA folding. (**a**) Splicing assay on mRNA, isolated from 293T cells, 48 h post-transfection with pNL4-3 HIV-1 WT or HIV-1 HA/FLAG-Vpr plasmids. This mRNA was reverse transcribed into cDNA and used as input material, together with primers p1–10, described in Supplement 7. Graph shows expression of unspliced (US), singly spliced (SS) and multiple spliced (MS) mRNAs relative to WT levels, normalized for reference gene expression and corrected for total HIV-1 RNA. Error bars indicate the standard error of the mean (SEM). Wilcoxon signed-rank test, N = 8, p = 0.007813 (p1 + 2), 0.007813 (p5 + 6), 0.007813 (p3 + 8), 0.007813 (p2 + 8), 0.007813 (p3 + 9), 0.4609 (p7 + 9). (**b**) Ratio of average Cq values of duplo PCR reactions (non-normalized data) obtained using total HIV-1 mRNA primers p1 + p10 and UBC FWD/REV primer set, N = 7 on cDNA from total cellular RNA of 293T cells transfected with pNL4-3 WT or HA/FLAG-Vpr plasmids, Mann-Whitney U test, p = 0,1282. (**c**) *In silico* mRNA folding predictions of NL4-3 HIV-1 WT or HA/FLAG-Vpr genomic region p5041–9017, using Genebee RNA secondary structure prediction algorithm. The circle specifies the region where predicted folding is altered, numbers indicate the position in the NL4-3 genome, starting from the 5′ LTR sequence. (**d**) *In silico* predictions of full NL4-3 HIV-1 WT or HA/FLAG-Vpr genome mRNA folding structure, using PPfold3.1 bioinformatics tool. The rectangle zoom-out and arrows specify the region where predicted folding is altered.

Since reduced viral production could be caused by oversplicing of genomic RNA^8^,^9^, we measured the expression levels of the major HIV-1 mRNA species by quantitative PCR (RT-qPCR) (Fig. 3a). We used primer sets to measure either unspliced (US), singly spliced (SS) and multiple spliced (MS) mRNA and one primer set to quantify total HIV-1 RNA. Results were expressed relative to WT values as described in Materials and Methods. We found significantly less US and SS mRNA expressed from HIV-1 HA/FLAG-Vpr, together with less Nef-2 expression (MS). We verified that the total HIV-1 RNA expression (also determined by transfection efficiency) was not affected (Fig. 3b), by comparing expression values of total HIV-1 RNA between untagged (WT) and HA/FLAG-Vpr. We compared the splicing in the VPR^ins20^, VPR^ins21^ and VPR^scr^ mutants and found, in accordance with unaffected viral production and infection, no difference compared to WT (Supplement 5). In conclusion, the observed defect of the HA/FLAG-Vpr correlates to altered mRNA splicing. This oversplicing of RNA will result in relatively reduced levels of Gag/Pol/Env major structural proteins, which is probably the underlying mechanism for the inability of this mutant virus to produce viral progeny^5^. Of note, the HSA marker is expressed from MS mRNA, explaining why this expression is not reduced in the 293T cells transfected with proviral HIV-1 HA/FLAG-Vpr (Fig. 2c).

### Vpr N terminus insertions that affect splicing alter RNA folding simulation

To explore the cause of oversplicing in the HA/FLAG-Vpr mutant, we first checked the integrity of known splice donor and acceptor sites, and of known splicing regulatory sequences^7^ and found these not to be affected by inserting the tags. Subsequently, we analyzed whether the mRNA folding could be affected. We compared folding of all mutant constructs with the WT virus. We used the Genebee web-based platform^21^ to simulate the folding of the HIV-1 genomic sequence. Due to size limits of the RNA secondary structure prediction algorithm, 8 kb genomic fragments of the HIV-1 NL4-3 strain were sequentially included in the algorithm to identify the differentially folded genomic region. Finally, as shown in Fig. 3c, we simulated the p5041–9017 region (WT numbering), encoding Vif-Env, and found that the HA/FLAG-Vpr folding but not that of the other VPR mutants deviated significantly from that of the WT sequence in the genomic region p5000–7000, a region rich in splice sites and splicing regulatory elements^22^. Comparative predictions of the full HIV-1 genome folding with the PPfold3.1 bioinformatics tool^23^ confirmed this observation (Fig. 3d). Together, these data suggest that mRNA folding is affected by the inserted tag, which could cause skewed viral mRNA splicing and the resulting block in viral production.

### HA/His_6_-Vpr as a functional alternative

Besides HA and FLAG, other small tags such as Myc, His_6_ and V5 are successfully used for tandem affinity purification. In order to find a functional substitute for HA/FLAG tandem tagged Vpr, we explored a variety of combinations including orientations with these five small tags via simulations with the Genebee platform.

**Figure 4:**
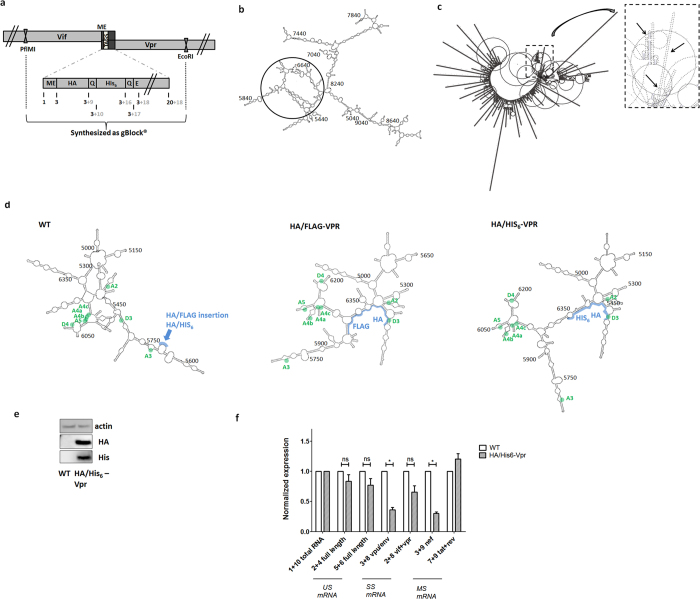
HIV-1 HA/His_6_-Vpr as a valid alternative. (**a**) Strategy used to construct HA/His6-Vpr starting from sequence verified gBlocks^®^. This was cloned in the pNL4-3 HIV HSA plasmid using the previously described cloning strategy (Supplement 1s). Numbers indicate amino acid position of Vpr (black), and number of amino acids added by tagging (grey). (**b**) *In silico* mRNA folding predictions of NL4-3 HIV-1 HA/His6-Vpr genomic region p5041–9017, using Genebee RNA secondary structure prediction algorithm. The circle specifies the region where predicted folding is altered, compared to the WT NL4-3 HIV-1 predicted folding, numbers indicate the position in the NL4-3 genome, starting from the 5′ LTR sequence. (**c**) *In silico* predictions of full HIV-1 HA/His6-Vpr genome mRNA folding structure, using PPfold3.1 bioinformatics tool. The rectangle zoom-out and arrows specify the region where predicted folding is altered. (**d**) *In silico* mRNA folding predictions of NL4-3 HIV-1 WT, HIV-1 HA/FLAG-Vpr and HIV-1 HA/His6-Vpr genomic region p5000–6500 (or extended with tags length), using Genebee. Location of 5′ and 3′ ss is indicated as green dots, based on^7^. The position of the inserted tags within the constructs is highlighted with a blue line. (**e**) Western blot for HA and His_6_ expression of pNL4-3 WT HIV-1 or pNL4-3 HIV-1 HA/His_6_-Vpr plasmid transfected 293T cells, 48h post-transfection. 10 μg of protein was loaded and β-actin was stained as loading control. (**f**) Splicing of mRNA, isolated from 293T cells, 48 h post-transfection with pNL4-3 WT HIV-1 or pNL4-3 HIV-1 HA/His_6_-Vpr plasmids, as described in Fig. 3a. Graph shows expression of unspliced (US), singly spliced (SS) and multiple spliced (MS) mRNA relative to WT levels, normalized for reference gene expression and corrected for total HIV-1 RNA. Error bars indicate the standard error of the mean (SEM).Wilcoxon signed-rank test, N = 6, p = 0.2188 (p2 + 4), 0.1563 (p5 + 6), 0.03125 (p3 + 8), 0.0625 (p2 + 8), 0.03125 (p3 + 9), 0.09375 (p7 + 9).

We found the HIV-1 HA/His_6_-Vpr virus (tags shown in Fig. 4a) to be the most valid option, with a predicted RNA folding structure (Fig. 4b,c) most resembling the WT architecture. Next we looked more specifically at the stem structures in region p5000–6500 in the NL4-3 plasmid, encompassing 3′ ss A2, A3, A4a-b-c, A5 and 5′ ss D3 (Fig. 4d). Remarkably, it appears that the 5′ ss D3 was captured in a stem structure with HA in both HA/FLAG-Vpr and HA/His_6_-Vpr virus. Also, supplementary stem structures were introduced between 3′ ss A4c-a-b that could affect splicing to these sites. Additionally, in both tagged constructs, the relative orientation of 3′ ss A3 and 5′ ss D4 appears to have changed in this model. These latter changes are however different between both constructs, suggesting splicing profiles may differ.

Confirming the folding predictions, functional tests indeed showed that viral production was not affected (Supplement 3) and also expression of the tags could be demonstrated (Fig. 4e). Merely, a modest effect on viral splicing was seen (Fig. 4f), characterized by decreased Vpu/Env and Nef transcript expression compared to untagged WT virus.

**Figure 5:**
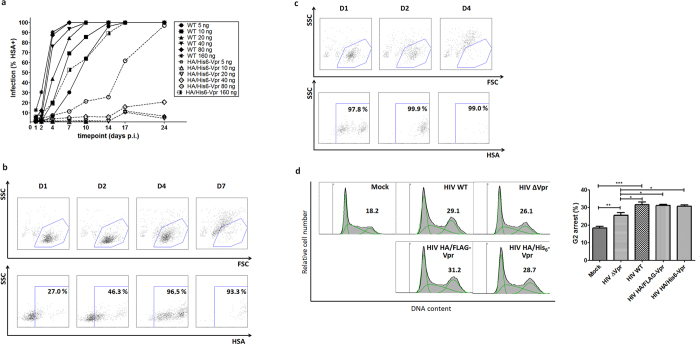
HIV-1 HA/His_6_-Vpr functional testing. (**a**) Replication curves of HIV-1 HA/His_6_-Vpr and WT HIV-1 after infection of SupT1 with 5–160 ng p24. Infection is measured by HSA expression, at several days post-infection (p.i.). WT replication is presented as solid lines; HA/His_6_-Vpr as dashed lines. (**b**) Increased viral input infection experiment in SupT1, using 160 ng p24 HIV-1 HA/His_6_-Vpr to monitor induction of cell death. Upper dot plots show forward (FSC) and side scatter (SSC), lower plots show HSA staining versus SSC for cells gated in the live scatter gate shown in the upper dot plots. Figures indicate percentage of cells staining positively with the APC labelled antibody. (**c**) SupT1 infection with VSV-G pseudotyped HA/His_6_-Vpr virus, 160 ng p24 virus. Upper dot plots show forward (FSC) and side scatter (SSC), lower plots show HSA staining versus SSC for cells gated in the live scatter gate shown in the upper dot plots. Figures indicate percentage of cells staining positively with the APC labelled antibody, gated on live cells as shown. (**d**) Cell cycle analysis on pNL4-3-transfected 293T cells 48 h post-transfection. 293T cells were transfected with the proviral constructs or Mock transfected, as indicated. Data were analyzed with the FlowJo cell cycle analysis platform on flow cytometry data of DNA staining, applying the Dean-Jett-Fox model. Left panel: histograms from a representative experiment. Separate green curves in the shaded histogram illustrate the fraction of cells in G1, S and G2 phase of the cell cycle. The percentage of cells in G2 phase (represented by green curve covering highest DNA content) is shown for each sample. Right panel: percentage of cells in G2 phase induced by the indicated constructs or Mock transfection. Error bars indicate standard deviation. Mann-Whitney U test comparing samples. N = 8 (Mock), N = 6 (HIV WT/HIV ΔVpr) or N = 5 (HIV HA/Flag-His_6_-Vpr). Mock-HIV WT p = 0.0007, Mock-HIV ΔVpr p = 0.0062, HIV WT–HIV ΔVpr p = 0.0303, HIV ΔVpr-HIV HA/FLAG-Vpr p = 0.0159, HIV ΔVpr-HIV HA/His_6_-Vpr p = 0.0317.

Viral titration infection on SupT1 cells (Fig. 5a and Supplement 6) showed reduced infectivity and replication of HIV-1 HA/His_6_-Vpr compared to the untagged WT virus, however to a lesser degree than the reduction seen with HIV-1 HA/FLAG-Vpr (compare to Fig. 2c). Upon adding higher amounts of input virus, efficient replication was initiated. These infection data are in line with the decreased amounts of Env and Nef transcripts, most likely responsible for reduced infectivity of the progeny virus.

To further characterize the functionality of the HIV-1 HA/His_6_-Vpr, we monitored both cytotoxic and cytostatic functions of Vpr. We found that, when sufficient infection levels were reached, cell death could be induced by replicating HA/His_6_-Vpr virus (Fig. 5b). VSV-G pseudotyping accelerated infection and Vpr cytopathicity (Fig. 5c). This would allow for single round infection in cells non-permissive for Vif deleted viruses. Cell cycle analysis of transfected 293T cells (Fig. 5d) confirms that N-terminal tagging does not affect the cytostatic function of Vpr. As a control in this cell cycle experiments, HIV-1 ΔVpr (HIV-1 HSA-IRES-Nef with stop codons in all 3 reading frames in the N-terminus of Vpr, after the N-terminal overlap with the Vif reading frame), not expressing Vpr was used^24^. Together, these data show the HIV-1 HA/His6-Vpr can be used to study Vpr function in the context of a replication competent virus.

## Discussion

We present the detrimental effect of HA/FLAG-Vpr tagging on viral production and infection, correlating with viral mRNA oversplicing. As a functional alternative, we developed the HIV-1 HA/His_6_-Vpr virus to be used in Vpr interaction studies when the use of replication competent virus is preferred over the use of single protein overexpression.

We started this study by comparing N- versus C-terminal tagging of Vpr for preservation of the cytopathicity by Vpr. Both strategies have their drawbacks for tagging, given the known domain-function relationships of the termini^25^. N-terminal modification might impede Vpr’s nuclear localization/import and infection of non-dividing cells^26^ or transactivation of transcription^27^. G2 arrest on the other hand, is mediated by the C-terminal α-helical domain^25^, although hydrophobic residues in the first N-terminal α-helix are also required to induce cell cycle arrest^28^. The cell death inducing domains of Vpr appear to be more dispersed throughout the protein and functionally depend on nuclear localization, but not on G2 arrest^25^.

In this study we found that C-terminal, but not N-terminal tagging, affected cytopathic function of Vpr. Therefore, the N-terminus of Vpr was tagged in HIV-1. Inevitably, introduction of N-terminal tags to Vpr within HIV-1 also modifies the C-terminus of Vif. This has no effect on infection in permissive SupT1 cells, as these do not require Vif expression to counteract APOBEC3G restriction^20^.

The ablated viral production and replication in both SupT1 and CEM CD4^+^ T cell lines observed with HIV-1 HA/FLAG-Vpr, was associated with a significant reduction in US and SS mRNA, characteristic for oversplicing. Madsen and Stoltzfus^8^,^9^ reported that oversplicing of HIV-1 genomic mRNA impedes viral production. Their studies show that mutational regulation of downstream 5′ splice donor site strength, or that of the exonic splicing silencer ESSV, can enhance the usage of suboptimal upstream 3′ splice acceptor sites A1 or A2, resulting in oversplicing and defective replication. The integrity of known splice sites and regulatory sequences^7^ is not affected by insertion of the HA/FLAG tags. Moreover, the reduction on viral production we observe is much more dramatic than that described in these and other studies^8^,^9^,^13^,^29^.

In our study, additional mutants produced to assess the genotype-phenotype correlation (either a N-terminal fragment of the original HA tag of 21 or 21 bp length inserted, or a 60 bp scrambled region at the same site to replace the wild-type sequence) did not show oversplicing nor defects in viral production, infection or replication. From these results we could conclude that the observed phenotype of HIV-1 HA/FLAG-Vpr was not due to disturbance of the Vif or Vpr reading frames and to be sequence specific.

We propose that an aberrant RNA folding structure is underlying the observed phenotype. Indeed, studies in eukaryotes have demonstrated the importance of mRNA folding in regulating primary transcript splicing. Secondary mRNA structures can inhibit or promote binding of spliceosomal elements to the pre-mRNA, or can bring important sequences into closer proximity to enhance efficiency of splicing (reviewed in^30^). Also in viruses, like the Duck hepatitis B virus^31^ or Rous sarcoma virus^32^, well-structured RNA sequences have been described that suppress splicing. In adenovirus, an intronic hairpin structure approximates crucial splice sites to improve splicing efficiency^33^. HIV-1 mutational and phylogenetic studies have similarly demonstrated the existence of local secondary mRNA structures and their importance for HIV-1 genomic mRNA splicing^34^,^35^. This balanced expression of unspliced and singly/multiple spliced mRNA is in turn decisive for efficient HIV-1 replication, as we and others demonstrate^8^,^9^.

Unlike local RNA structures, HIV-1 long range RNA-RNA interactions and higher order, tertiary structures are less well documented, and biological data on this matter are lacking. There is some *in vitro* evidence for the existence of a long range pseudoknot in the 5′-untranslated and Matrix coding regions of HIV-1 genomic RNA^36^, as confirmed via the SHAPE (selective 2′-hydroxyl acylation analyzed by primer extension)-based study on the architecture of the full NL4-3 HIV-1 RNA genome^37^. An *in silico* study by Assis^38^, demonstrated the covariance of single nucleotide mutations in HIV-1 evolution, what suggests a strong epistatic selection of the HIV-1 tertiary structure. Also more recently, a comparative model for the higher order structure of the HIV-1 genome was published by Süküsd and colleagues^39^, revealing a conserved core domain, formed by a set of long distance interactions with a particular high frequency of compensatory base changes.

Our *in silico* results suggest that the aberrant viral production and replication, observed after N-terminal tagging of Vpr, is likely due to an effect on tertiary mRNA folding of the HIV-1 transcripts. However, one should bear in mind that mRNA folding simulates only folding potentials, making it difficult to assess structure-function correlations.

Nevertheless, the structural impact of the insertions were experimentally shown to be sequence specific: simulation showed that aberrant nucleotide bridging arose between the inserted HA/FLAG-Vpr tags and both up/downstream mRNA sequences changed folding. Mutants VPR^ins20^, VPR^ins21^ and VPR^scr^ support the validity of the simulations as their predicted structures fitted more with the WT structure and no functional defects could be observed.

As an alternative for tagging we propose the HA/His_6_-Vpr virus. This does not have the same simulated folding alterations as the HA/FLAG-Vpr virus, yet still showed more moderate disturbed splicing, with reduced Env/Vpu and Nef mRNA expression levels. The latter can explain the reduced infection rate observed, since both Env and Nef expression are known to enhance viral infectivity^40^. Still, HIV-1 HA/His_6_-Vpr is suitable for studies on Vpr function in a replicating virus. Published studies with tagged Vpr in HIV-1 replication generally express Vpr *in trans*, limiting the replication to a single round of infection. Yet a limited number of studies describe the introduction of single Vpr protein tags HA or FLAG in the proviral DNA^41^,^42^. Unfortunately these studies do not assess the impact of this tagging on viral production, infectivity and resulting fitness.

Understanding the dynamics and interactions of HIV-1 mRNA is important to further decipher the virus and might aid to develop new therapeutic strategies. This knowledge could be applied to target and modify mRNA folding/splicing of HIV-1. Indeed, previous studies demonstrated the power of controlling HIV-1 alternative splicing and multiplication using antisense derivatives of U7 small nuclear RNA^43^ or small molecules IDC16 and 8-Azaguanine^44^,^45^. Similarly, ABX464 – a small molecule inhibiting Rev-mediated export of US RNA – is able to compromise HIV-1 replication in a humanized mouse model which now awaits confirmation in a Phase IIa clinical trial^46^.

It is becoming more and more clear that conservation of the tertiary structure of HIV-1 is important for efficient viral biogenesis. This offers new therapeutic possibilities, aiming to support current therapy regimens. As the mRNA folding is encoded within the viral genetic code, *in vitro* manipulations of highly conserved regions can therefore interfere with proper genomic folding. This should be considered when tagging proteins for interaction studies.

## Materials and Methods

### Molecular cloning pLZRS and pNL4-3 constructs

Full experimental details are described in Supplement 1

### Cell culture

Human embryonic kidney 293T (DZSM, Braunschweig, Germany), Phoenix-Amphotropic packaging (Phoenix A) cells (kind gift from Dr. P. Achacoso and Dr. G.P. Nolan, Stanford University School of Medicine, Stanford, CA)^47^ and SupT1 (AIDS Research and Reference Reagent Program, National Institutes of Health, Bethesda, MD) were cultured at 37 °C in a 7% CO_2_ (vol/vol) humidified atmosphere, in IMDM complete medium (IMDMc): Iscove’s modified Dulbecco’s medium (Life Technologies, Merelbeke, Belgium) supplemented with 10% (vol/vol) fetal bovine serum (FBS; Hyclone, Thermofisher Scientific, Waltham, MA) and 2 mM L-glutamine (Life Technologies). CEM-GFP cells were obtained through the NIH AIDS Reagent Program, Division of AIDS, NIAID, NIH (originating from Dr. Jacques Corbeil, Infectious Diseases Research Center Quebec^48^) and cultured at 37 °C in a 5% CO_2_ (vol/vol) humidified atmosphere, in RPMI 1640 medium with 10% (vol/vol) FBS and 2 mM L-glutamine.

### Cell cycle analysis

Nuclear DNA content was assessed in 293T according to the protocol of Wen and colleagues^49^. Before jetPEI^®^ DNA transfection, 293T cells were seeded at 4 × 10^5 ^cells/4 ml IMDM complete (containing 10% (vol/vol) FBS, 100 U/mL penicillin and 100 g/mL streptomycin (both from Life Technologies)) in 6 well cell culture plates (Corning, New York, NY). The next day, 4 μg plasmids were transfected to the producer cells as instructed by supplier (Polyplus, New York). 48 h post-transfection, cells were detached and analyzed for DNA content: 2 × 10^5^ cells were suspended in 200 μl PBS and 200 μl cell cycle staining buffer (see ref. 49) and incubated at 37 °C for 20 min. Transfection efficiency was verified by dNGFR and HSA markers. pNL4-3 transfection was comparable between different constructs and reached 90%. pLZRS transfection was below 50% and therefore cells were sorted using a BD FACSAria III cell sorter.

### LZRS retroviral production and transduction

Retroviral particles were produced in PhoenixA cells as described before^50^; supernatant was harvested 48 h and 72 h post-transfection. For transduction, 5 × 10^4^ SupT1 cells were mixed and spinoculated with 100 μl retroviral supernatant and DOTAP as described previously^50^. At time points d2 (48 h), d4 and d7, cells were counted and stained for dNGFR expression.

### HIV-1 production, Reverse Transcriptase assay and HIV-1 infection SupT1 and CEM-GFP

7.5 × 10^5^ 293T cells were seeded in 6 cm culture dishes in 6 ml IMDMc. Cells were transfected the next day with 5 μg plasmid DNA using jetPEI as described for cell cycle analysis. Virus was harvested 48 and 72 h post-transfection and frozen at −80 °C. An RT assay was performed as previously described^51^. For infection, 5 × 10^4^ cells were mixed and spinoculated with 100 μl viral dilution as described previously^50^. At the described time points, infection was quantified using a GFP or HSA marker.

### Flow cytometry

Monoclonal antibodies were used to stain HSA (CD24-APC, clone M1/69, Biolegend, San Diego, CA) or dNGFR (NGFR-APC, clone ME20.4, Chromaprobe, Maryland Heights, MO, USA). Data were acquired on a Miltenyi MACSQuant^®^ Analyzer or BD FACSCalibur flow cytometer (for cell cycle analysis) and analyzed with FlowJo analysis software (FlowJo LLC, Ashland, USA).

### Splicing RT-qPCR

HIV-1 mRNA species were quantified 48 h after pNL4-3 transfection of 293T cells, as described for HIV-1 production. mRNA was isolated, DNaseI treated and converted into cDNA as described previously^50^. Primers p1-p9 combinations for quantification of spliced and unspliced mRNA were adopted from Houzet and colleagues^52^. Primer p10 was developed in-house to quantify total mRNA (see Supplement 7). qPCR reactions were run on a LightCycler^®^ 480 Real-Time PCR system as described previously^50^. RT-qPCR data were generated and analyzed using the MIQE guidelines and by using qBase data analysis software^53^. Results were normalized using reference genes ubiquitin C (UBC), TATA Box Binding Protein (TBP) and tyrosine 3-monooxygenase/tryptophan 5-monooxygenase activation protein zeta (YWHAZ) to correct for sample to sample variation in cDNA yield. Next, expression levels were scaled to the WT sample for each primer set in qBase. This allows for the comparison of the expression levels of the different target genes between WT and mutants. Finally, these expression levels were corrected for total HIV mRNA (eg. affected by transfection efficiency), as measured by primer combination 1 + 10 in our experiments. To this end, expression levels of targets measuring US, SS and MS mRNA were divided by the relative expression value of primer set 1 + 10 measured for that particular construct.

### Western blotting

Transfected cells were lysed in 1X Laemmli buffer at 120 μl/10^6^ cells and sheared to reduce viscosity. 10 μg of protein lysate was combined with 4X Bolt^®^ LDS Sample Buffer (Life Technologies) and loaded to a Bolt™ 4–12% Bis-Tris Plus Gel and run with MES SDS Running buffer (Bolt). After gel electrophoresis, proteins were transferred to a PVDF membrane, which was blocked (TBS, 5% skim milk, 0.075% Tween-20) for 4 h and stained overnight at 4 °C with monoclonal antibodies against HA (1/50000; clone 2–2.2.14, Life technologies), FLAG (1/125000; clone M2, Sigma-Aldrich), His_6_ (1/2000; clone HIS.H8, Life Technologies) or β-actin (1/2500; clone BA3R, Life Technologies). Secondary staining was performed with HRP-linked sheep-anti-mouse antibody (1/2000; NA931V, GE Healthcare Life Sciences, Little Chalfont, UK). Read-out was performed with Pierce™ ECL Western Blotting Substrate (Life Technologies) on an ImageQuant™ LAS 4000 imager (GE Healthcare Life Sciences).

### Prediction of HIV-1 RNA Secondary Structure by Folding analysis

The Genebee Molecular Biology Server^21^ was used to predict RNA folding of HIV-1 genomic fragment p5041–9017.

The PPfold (v.3.1.1) algorithm was used to predict HIV-1 RNA secondary structure of the full genome^23^. PPfold was run with default settings using the High Performance Computing clusters of UGent. Folding results were stored in cheat table (.ct) files, after which the VARNA java application (v.3.93) was applied to visualize the RNA secondary structures predicted by PPfold^54^. The NAView layout algorithm was implemented for visualization.

### Calculations and statistics

RT-qPCR data were normalized using qBasePLUS software 2.0 (Biogazelle, Zwijnaarde, Belgium) and the resulting “calibrated normalized relative quantities” (CNRQ) values and standard errors of this value (SE CNRQ) were then further analyzed with GraphPad Prism version 5.04 (GraphPad Software, San Diego, California, USA). RT activity of produced HIV-1 virus was calculated from Cq values with Excel 2007 (Microsoft, Redmond, WA)^51^. GraphPad was used to calculate and plot means and standard error of the mean (SEM), generate data plots and perform statistical tests as described for the respective figures.

## Additional Information

**How to cite this article**: Baeyens, A. *et al*. HIV-1 Vpr N-terminal tagging affects alternative splicing of the viral genome. *Sci. Rep.* **6**, 34573; doi: 10.1038/srep34573 (2016).

## Supplementary Material

Supplementary Information
