# Supplementary material for: HIV-1 Vpr N-terminal tagging affects alternative splicing of the viral genome
Source: Sci Rep. 2016 Oct 10;6:34573. doi: 10.1038/srep34573 (PMC5056386; doi:10.1038/srep34573)
Supplement: Supplementary Information [file srep34573-s1.pdf]

HIV-1 Vpr N-terminal tagging affects alternative splicing of the viral genome

Ann Baeyens1, Evelien Naessens1, Anouk Van Nuffel1, Karin E Weening1, Anne-Marie Reilly1, Eva Claeys1, Wim Trypsteen2, Linos Vandekerckhove2, Sven Eyckerman3,4, Kris Gevaert3,4, Bruno Verhasselt1,*

Supplementary information

Supplement 1. Molecular cloning pLZRS and pNL4-3 constructs

Retroviral vector pLZRS cloning

The NL4-3 Vpr sequence [1] was amplified by PCR (Platinum® Pfx DNA polymerase, Thermo Fisher Scientific, Waltham, MA) as instructed by the supplier with primers FWD C and REV N or with tailed primers FWD N (containing N-terminal tags) or REV C (containing C-terminal tags); together with primers REV N or FWD C, respectively (primer sequences enclosed in Supplement 7). Cloned PCR products were verified by sequencing and transferred to the retroviral vector pLZRS [2] using restriction sites BamHI and XhoI, as were added by FWD C/N and REV C/N primers. Vpr proteins are expressed from a bicistronic mRNA (thanks to the IRES sequence), also encoding dNGFR as a separate marker protein to identify construct expressing cells.

pNL4-3 HIV-1 HA/FLAG-Vpr cloning

The pNL4-3-HSA-IRES-Nef WT plasmid (kindly provided by Dr. M.J. Tremblay, Faculté de Médecine, Université Laval, Québec, Canada) [3] was used as a parental vector for cloning. This plamid encodes a replication competent HIV-1 virus, modified in the nef reading frame to express nef-IRES-HSA, thus both Nef and the marker protein HSA, what allows to identify infected cells by flow cytometry. The N-terminal region of Vpr was subcloned as a 440 bp PflMI-EcoRI-fragment containing a fragment of both Vif and Vpr. To this fragment tags were added using tailed primers. Two PCR products were created using primer combinations FWD1/REV1 and FWD N/REV 2. Primer FWD N incorporates the HA/FLAG tags, which are preceded by Met-Glu (ME) and are separated by Gln (Q) linkers. These products were combined in a third PCR reaction, using 50 ng of each purified PCR product and outlying primers FWD 1 and REV 2. The PCR program consisted of an initial denaturation of 2

minutes (min) at 94°C, next 10 cycles of amplification without primers: 30 seconds (s) at 94°C, 1 min at 57°C and 1 min at 68°C, followed by 30 cycles of amplification with primers and a final elongation step of 4 min at 68°C. The PCR product was sequenced and then cloned back to pNL4-3 to replace the untagged Vpr. Primer sequences can be found in Supplement 7.

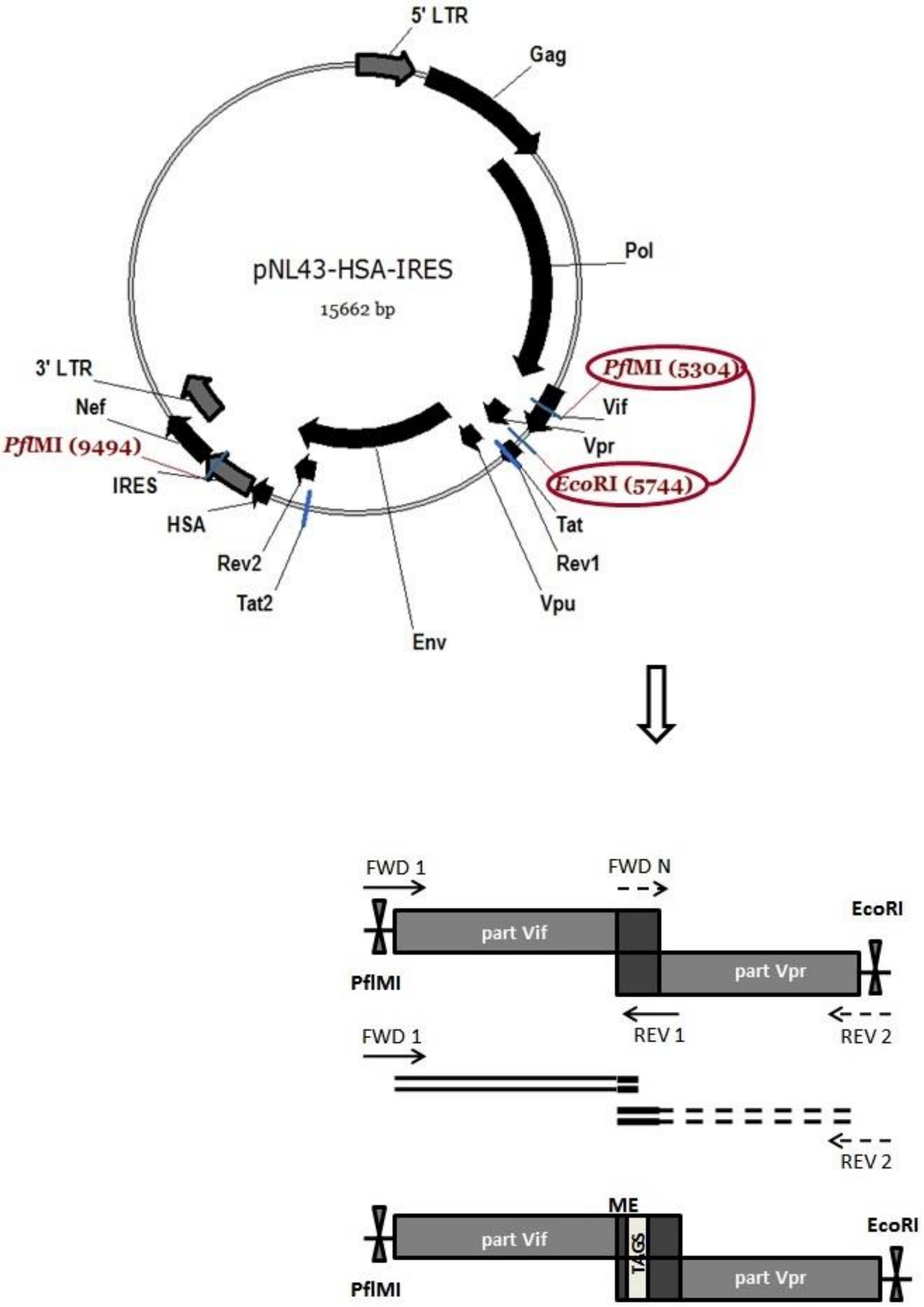

pNL4-3 HIV-1 VPR^{ins20}, VPR^{ins21} and VPR^{scr} cloning

The VPR mutants were created by PCR and restriction as was described for the HA/FLAG-Vpr mutant, and using the same PflMI-EcoRI subfragment as template as well as the same protocol for PCR. However, primer annealing temperatures (Ta) were adjusted depending on the primers used. Primer combinations and Ta are described in the table below. Primer sequences can be found in Supplement 7.

mutant	PCR step 1+2	Ta	primers	PCR step 3	Ta	primers
VPR^{ins20}	PCR 1	57°C	FWD 1	Nested PCR	55°C	FWD 1
			VPR^{ins20} REV 1			
	PCR 2	55°C	VPR^{ins20} FWD 2			VPR REV 2
			VPR REV 2			
VPR^{ins21}	PCR 1	57°C	FWD 1	Nested PCR	55°C	FWD 1
			VPR^{ins21} REV 1			
	PCR 2	55°C	VPR^{ins21} FWD 2			VPR REV 2
			VPR REV 2			
VPR^{scr}	PCR 1	57°C	FWD 1	Nested PCR	55°C	FWD 1
			VPR^{scr} REV 1			
	PCR 2	55°C	VPR^{scr} FWD 2			VPR REV 2
			VPR REV 2			

pNL4-3 HIV-1 HA/His$_6$-Vpr cloning

The pNL4-3 HA/His$_6$-Vpr cloning was started from a synthesized dsDNA gene fragment (gBlocks®, IDT), encoding the NL4-3 PflMI-EcoRI fragment with HA and His$_6$ tags. The sequence of the gBlocks® was verified and is enclosed in Supplement 7. This gene fragment was cloned in pNL4-3 as described for pNL4-3 HA/FLAG-Vpr cloning.

Supplement 2. HIV-1 HA/FLAG-Vpr replication in CEM-GFP.

Cells were infected with 40 ng (p24) WT HIV or 100 µl HA/FLAG-Vpr HIV (corresponding to 4-6 ng (p24)), as done for SupT1 (compare to Fig. 2E). Infection is quantified by GFP expression on different days (D1 to D16 as indicated) post-infection, as response to LTR transcriptional activity. Flow cytometry dot plots (GFP versus side scatter) from a representative experiment are shown (left). Figures in dot plots indicate percentage of GFP positive cells (in rectangular gate). These data (mean ± SEM of 2 experiments) is plotted over time (right).

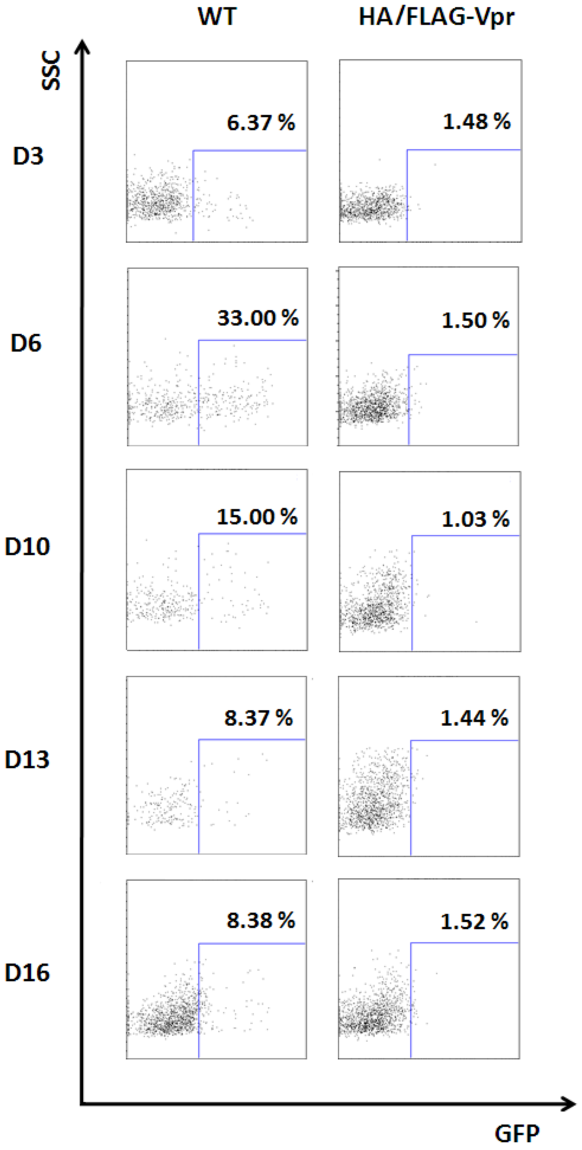

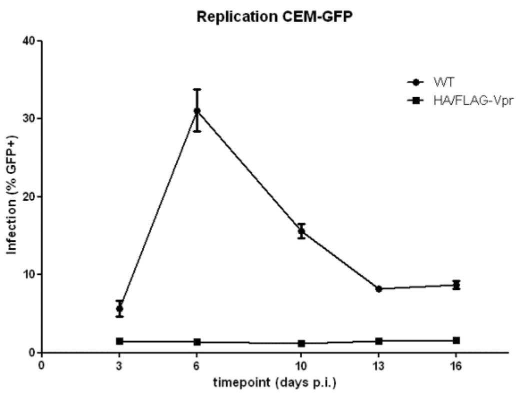

Supplement 3. Reverse Transcriptase (RT) activity of 293T-produced virus. 48 h post-transfection, viral supernatants was harvested and quantified for RT activity. Left panel: WT virus with HSA reporter, NL4-3 HA/FLAG-Vpr (also with HSA reporter or WT backbone) N=1. Right panel: NL4-3 WT and NL4-3 HA/His_6-Vpr (both HSA backbone, mean values ± SEM). N=5, Mann-Whitney U test, p=0.6905.

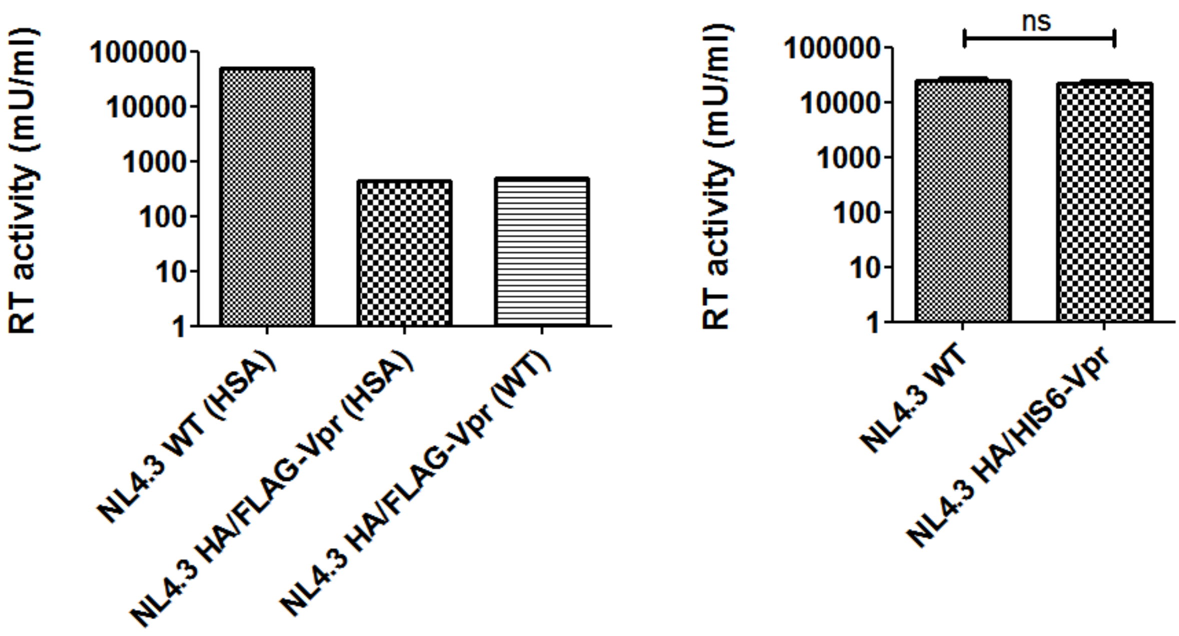

Supplement 4. VPR mutants Reverse Transcriptase (RT) activity and infectivity.

Viral supernatant was produced as described in Material and Methods and the average RT activity ± SEM of 3 experiments was plotted (upper panel). SupT1 cells were infected with 20 ng (p24) of virus and infection was assessed on day 1, 2, 4 and 7 (lower panels). For infection and replication, the average of 9 samples ± SEM is plotted.

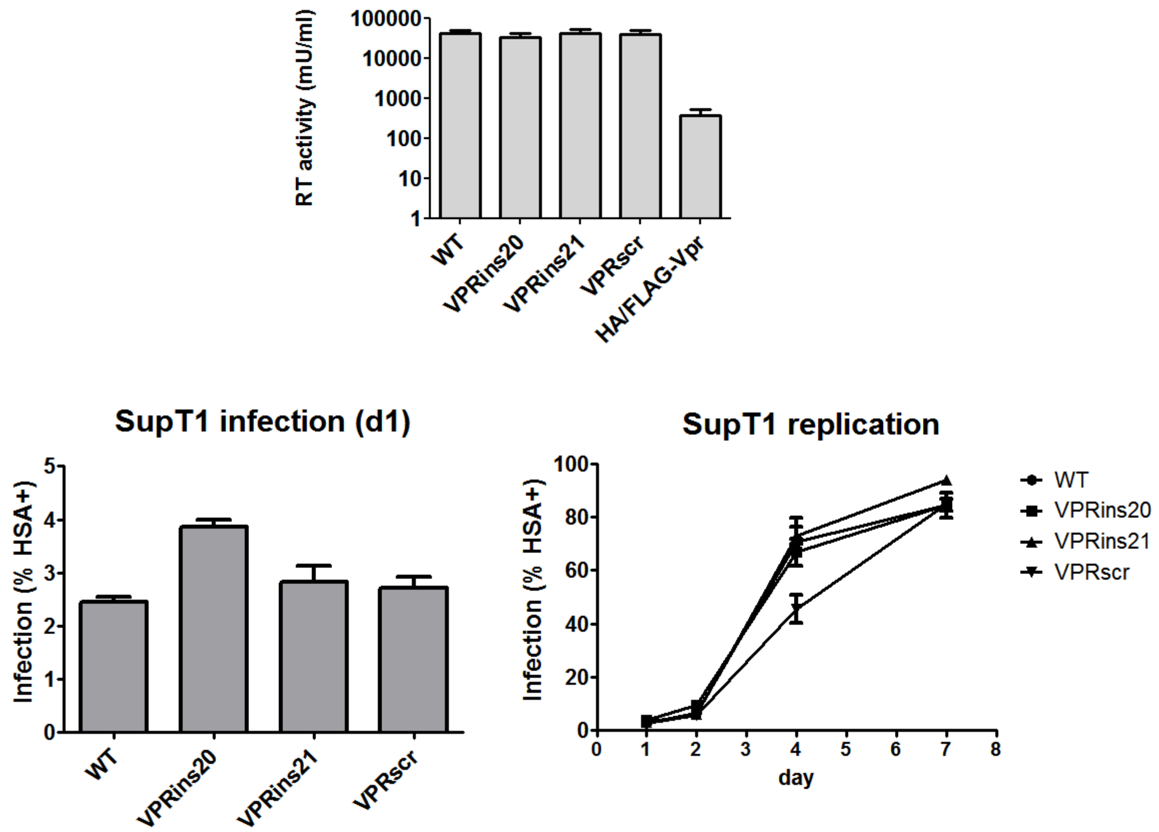

Supplement 5. VPR mutants splicing assay.

Splicing was assessed on RNA, isolated from transfected 293T cells, as described. Graph shows expression of unspliced (US), singly spliced (SS) and multiple spliced (MS) mRNA relative to average expression levels from all mutants and normalized for reference gene expression.

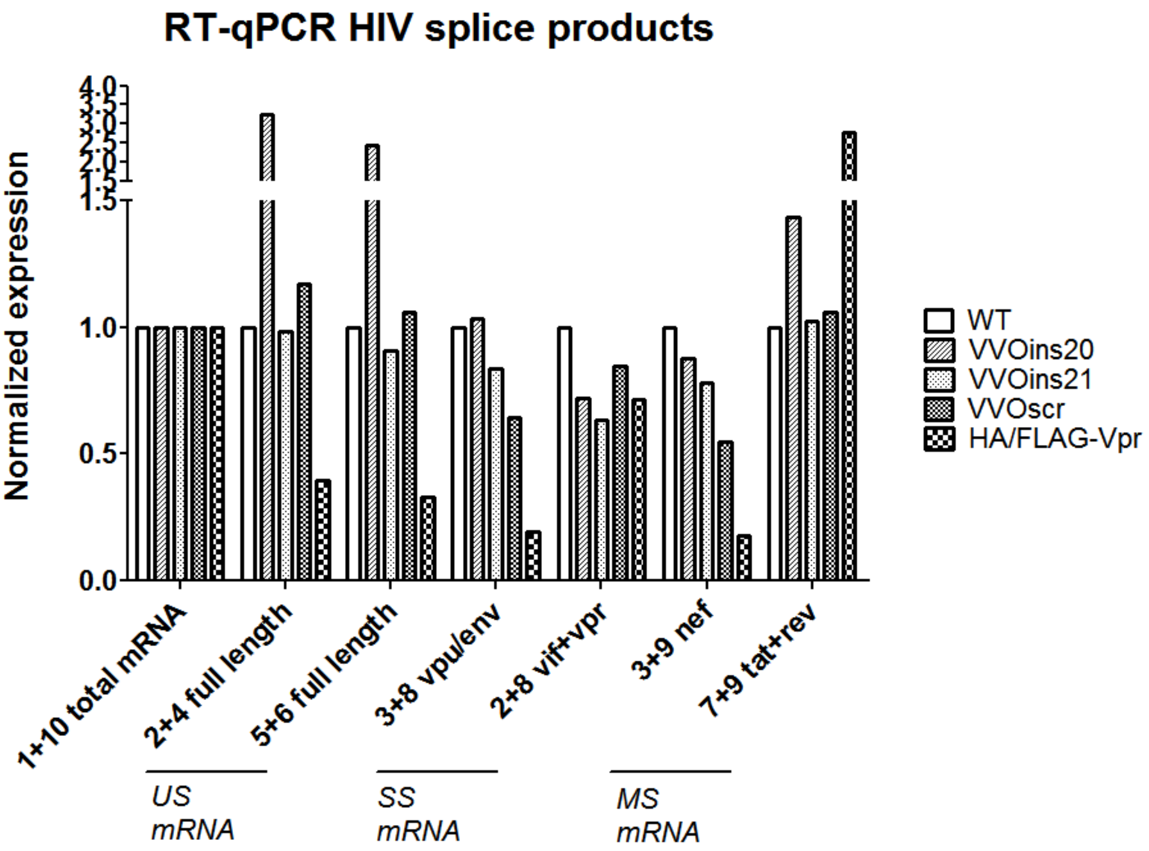

Supplement 6. HIV-1 HA/His$_6$-Vpr infection in SupT1. Infection measured 4 days post-infection by HSA-IRES-Nef marker expression from NL4-3 backbone, either WT (untagged Vpr) or HA/His$_6$-Vpr. Plot shows mean ± SEM, N=9, Mann-Whitney U test, p=0.0003.

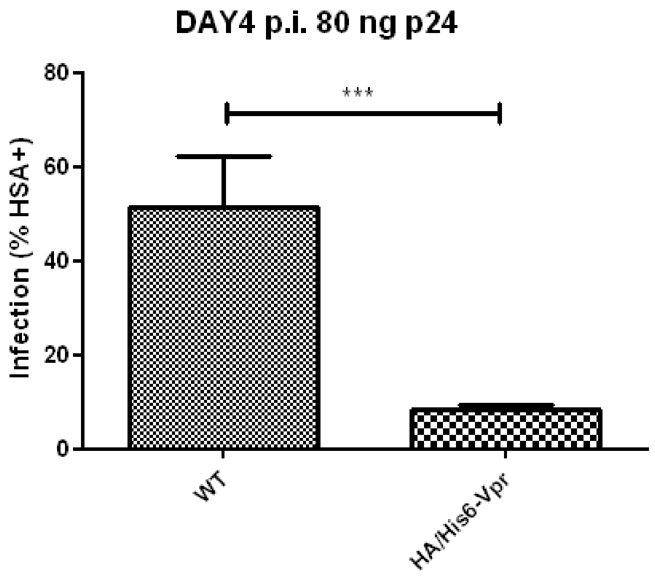

Supplement 7. Primers and gene fragment. Table with primers used for cloning and HIV splicing qPCR. Primer p1-p9 were adopted from Houzet and colleagues [4]; primer p10 and primers for reference genes were developed in-house. Primer p10 was developed to still bind the 3' LTR like the original p10 primer presented in [4]. The gBlocks® fragment (IDT) was used as a gene fragment for cloning, substituting PCR steps. The tag sequences used to encode HA, FLAG or His_6 are highlighted in bold.

**pLZRS Vpr N/C TAP cloning**	
**primer**	**sequence**
FWD N (**HA/FLAG**)	5'-AAGGATCCATGGAA**TACCCCTACGACGTCCCCGACTACGCCCAGGACTACAAGGACGACGACGACAAG**CAGGAACAAGCCCCAGAAGACCAA-3'
REV N	5'-TTCTCGAGTTGGCGCGCCAGTCTAGGATCTACTGGCTCC-3'
FWD C	5'-AAGGATCCCAGATGGAACAAGCCCCAGAAGACCC-3'
REV C (**HA/FLAG**)	5'-TTCTCGAGCTA**GGCGTAGTCGGGGACGTCGTAGGGGTA**TTGC**TTGTCGTCGTCGTCCTTGTAGTC**TTGGGATCTACTGGCTCCATTTCTTG-3'
**pNL4.3 HA/FLAG-Vpr cloning**	
**primer**	**sequence**
FWD 1	5'-GGGTCAGGGAGTCTCCATAGAATGG-3'
REV 1	5'-TCGTAGGGGTATTCCATCTGTCCTCTG-3'
REV 2	5'-ATAAACAGCAGTTGTTGCAGAATTC-3'
**pNL4.3 VVO$^{ins20/21}$ and VVO^{scr} cloning**	
**primer**	**sequence**
VPR^{ins20} REV 1	5'-TCGGGGACGTCGTAGGGGTA-3'
VPR^{ins21} REV 1	5'-GTCGGGGACGTCGTAGGGGTA-3'
VPR^{ins20} FWD 2	5'-TACCCCTACGACGTCCCCGACAAGCCCCAGAAGACCAAGG-3'
VPR^{ins21} FWD 2	5'-TACCCCTACGACGTCCCCGACCAAGCCCCAGAAGACCAAGG-3'
VPR REV 2	5'-GAATTCTGCAACAACTGCTGTTTAT-3'
VPR^{scr} REV 1	5'-TCTAAAAGCTCTATCCGATGCGTTGACCGACAGCATGCAGTACCTGGGCGATTCGGATAATGACAGCACATCTGTCCTCTGTCAGTTTCCTAACACT-3'
VPR^{scr} FWD 2	5'-CATCGGATAGAGCTTTTAGAGGAACTTAAGAGTGA-3'

**HIV splicing qPCR**	
**primer**	**sequence**
p1	5'-GATTGGCAGAACTACACACCAGGGC-3'
p2	5'-GTGGAAAATCTCTAGCAGTGGCGC-3'
p3	5'-GAGGGGCGGCGACTGGAAGAA-3'
p4	5'-TCTTTCCCCCTGGCCTTAACCG-3'
p5	5'-TCAGCATTATCAGAAGGAGCCACC-3'
p6	5'-TCATCCATCCTATTTGTTCCTGAAG-3'
p7	5'-GAAGAAGCGGAGACAGCGACGAAG-3'
p8	5'-ACTATGGACCACACAACTATTGC-3'
p9	5'-CAAGCGGTGGTAGCTGAAGAG-3'
p10	5'-GCGGCTGTCAAACCTCCACTC-3'
UBC FWD	5'-ATTTGGGTCGCGGTTCTTG-3'
UBC REV	5'-TGCCTTGACATTCTCGATGGT-3'
TBP FWD	5'-CACGAACCACGGCACTGATT-3'
TBP REV	5'-TTTTCTTGCTGCCAGTCTGGAC-3'
YWHAZ FWD	5'-ACTTTTGGTACATTGTGGCTTCAA-3'
YWHAZ REV	5'-CCGCCAGGACAAACCAGTAT-3'
**pNL4.3 HA/His$_6$-Vpr cloning**	
**gBlocks®**	**sequence**
NL4.3 **HA/His6**-Vpr gBlocks® PflMI-EcoRI	5'-<br>AAACCATAGAATGGAGGAAAAAGAGATATAGCACACAAGTAGAC<br>CCCGACCTAACAGACCAACTAATTCATCTGCACTATTTTGATTGTT<br>TTTCAGAATCTGCTATAAGAAATACCATATTAGGACGTATAGTTA<br>GTCCTAGGTGTGAATATCAAGCAGGACATAACAAGGTAGGATCTC<br>TACAGTACTTGGCACTAGCAGCATTAATAAAACCAAAACAGATA<br>AAGCCACCTTTGCCTAGTGTTAGGAAACTGACAGAGGACAGATGG<br>AA**TACCCCTACGACGTCCCCGACTACGCC**CAG**CATCATCACC**<br>**ATCACCAC**CAGCAAGCCCCAGAAGACCAAGGGCCACAGAGGGA<br>GCCATACAATGAATGGACACTAGAGCTTTTAGAGGAACTTAAGAG<br>TGAAGCTGTTAGACATTTTCCTAGGATATGGCTCCATAACTTAGGA<br>CAACATATCTATGAAACTTACGGGGATACTTGGGCAGGAGTGGAA<br>GCCATAATAAGAATTCAAA-3'

HIV splicing qPCR	
**primer**	**sequence**
p1	5'-GATTGGCAGAACTACACACCAGGGC-3'
p2	5'-GTGGAAAATCTCTAGCAGTGGCGC-3'
p3	5'-GAGGGGCGGCGACTGGAAGAA-3'
p4	5'-TCTTTCCCCCTGGCCTTAACCG-3'
p5	5'-TCAGCATTATCAGAAGGAGCCACC-3'
p6	5'-TCATCCATCCTATTTGTTCCTGAAG-3'
p7	5'-GAAGAAGCGGAGACAGCGACGAAG-3'
p8	5'-ACTATGGACCACACAACTATTGC-3'
p9	5'-CAAGCGGTGGTAGCTGAAGAG-3'
p10	5'-GCGGCTGTCAAACCTCCACTC-3'
UBC FWD	5'-ATTTGGGTCGCGGTTCTTG-3'
UBC REV	5'-TGCCTTGACATTCTCGATGGT-3'
TBP FWD	5'-CACGAACCACGGCACTGATT-3'
TBP REV	5'-TTTTCTTGCTGCCAGTCTGGAC-3'
YWHAZ FWD	5'-ACTTTTGGTACATTGTGGCTTCAA-3'
YWHAZ REV	5'-CCGCCAGGACAAACCAGTAT-3'
**pNL4.3 HA/His$_6$-Vpr cloning**	
**gBlocks®**	**sequence**
NL4.3 **HA/His6**-Vpr gBlocks® PflMI-EcoRI	5'-<br>AAACCATAGAATGGAGGAAAAAGAGATATAGCA<br>CACAAGTAGACCCCGACCTAACAGACCAACTAA<br>TTCATCTGCACTATTTTGATTGTTTTTCAGAATCTG<br>CTATAAGAAATACCATATTAGGACGTATAGTTAG<br>TCCTAGGTGTGAATATCAAGCAGGACATAACAAG<br>GTAGGATCTCTACAGTACTTGGCACTAGCAGCAT<br>TAATAAAACCAAAACAGATAAAGCCACCTTTGC<br>CTAGTGTTAGGAAACTGACAGAGGACAGATGGA<br>A**TACCCCTACGACGTCCCCGACTACGCC**CAG**C**<br>**ATCATCACCATCACCAC**CAGCAAGCCCCAGAAG<br>ACCAAGGGCCACAGAGGGAGCCATACAATGAAT<br>GGACACTAGAGCTTTTAGAGGAACTTAAGAGTGA<br>AGCTGTTAGACATTTTCCTAGGATATGGCTCCATA<br>ACTTAGGACAACATATCTATGAAACTTACGGGGA<br>TACTTGGGCAGGAGTGGAAGCCATAATAAGAATT<br>CAAA-3'

References

1. Adachi, A., et al. Production of acquired immunodeficiency syndrome-associated retrovirus in human and nonhuman cells transfected with an infectious molecular clone. *J Virol.* **59,** 284-291 (1986).

2. Landi, A., et al. Genome-wide shRNA screening identifies host factors involved in early endocytic events for HIV-1-induced CD4 down-regulation. *Retrovirol.* **11,** doi: 10.1186/s12977-12014-10118-12974. (2014).

3. Imbeault, M., Lodge, R., Ouellet,M., Tremblay, M.J. Efficient magnetic bead-based separation of HIV-1-infected cells using an improved reporter virus system reveals that p53 up-regulation occurs exclusively in the virus-expressing cell population. *Virol.* **393,** 160-167 (2009).

4. Houzet, L., et al. HIV controls the selective packaging of genomic, spliced viral and cellular RNAs into virions through different mechanisms. *Nucleic Acids Res.* **35,** 2695-2704 (2007).
